# Clinical Cases

**Published:** 1899-05

**Authors:** Stuart Close

**Affiliations:** Brooklyn, N. Y.


### CLINICAL CASES.

Stuart Close, M. D., Brooklyn, N. Y.

Case I.—Boy aged three years. Light complexion, blue eyes. Circumcised one year ago. Operation was unskillfully performed, too much tissue having been removed. Remaining tissue of prepuce is loose, flabby, falls away from glans and with the frenum depends nearly half an inch below under side of penis. This loose, flabby tissue is inflamed, red, the skin and exposed mucous surface rather thickened and rough, and covered with an itching eruption of vesicles, which ooze moisture and form scabs.

The meatus is inflamed and covered with a scab, which covers and closes the meatus, and must be broken or picked off every time before micturition can be performed, causing great pain. He screams whenever this occurs. He is restless and irritable, unhappy and dissatisfied with everything, and is very cross. Wakes frequently at night, crying, with pain in abdomen, worse on right side, apparently from flatus.

Diarrhœa; yellow or brownish offensive stools, which are lumpy and contain undigested food, aggravated by eating sweets. He has been unsuccessfully treated for foregoing symptoms nearly four months by one of the "leading homœopathic physicians of Brooklyn," who has prescribed many remedies and used many local applications.

On April 25th, 1898, I prescribed Graph. 45M (Fincke), one dose dry, with Sac-lac. powders *quantum sufficit.* In one week improvement was manifest. Itching had ceased, vesicles had begun to disappear and redness and swelling were decreased. Not so much trouble at meatus. Diarrhœa had ceased, and he acted generally better. Two weeks from first prescription, eruption was all gone, and the child well. The following week an attack of measles began; pursued a normal course under a few doses of Pulsatilla, 200, and ended in about eight days. A slight return at that time of the eruption at the meatus was speedily removed by a second dose of Graph., 45M, and he has since remained in perfect health. Considerable contraction has taken place in the flabby preputial tissue, which is now but slightly pendant.

Case 2, March 8th, 1898.—Married woman, aged 36 years. Normal twin confinement in July last. Has borne three children in two years. Since getting up from last confinement has been able to walk or stand only with great suffering on account of a severe pain, shooting, burning and tingling like an electric shock, starting from balls of feet and extending through feet to ankles, and sometimes to knees; worse on left side. She feels no pain when walking barefoot or with stockings only, but even a loose shoe causes such pain that she cannot walk more than a block.

Hemorrhoids just before and during menses, when they feel full as if bursting, and sometimes bleed a little.

The patient came just at the close of my office hour, when I was in a hurry to get out, and I violated Hahnemannian principles by asking a leading question and making a "snapshot" prescription. I did not examine the feet, but asked her if the balls of the feet were calloused. She replied affirmatively, and I gave her Antimon-crud. 200, telling her to call in a week. A week later she returned and reported "no change." On this occasion I examined her feet and found that there was no callous, and that objectively the feet were in good condition. Standing on her bare feet with the weight of the body borne mostly on the heels, she felt no pain, but deep pressure on the ball of the foot over the digital branches of the plantar nerves caused the cutting, burning, tingling pains. I also learned that she had a laming sore pain, with stiffness, in the sacral region, which made turning over in bed difficult, and was worse when she had been much on her feet. On rising in morning sensation in soles as if stepping on needles.

Eruption of suppurating pimples on chin before menses.

After study of the case I prescribed Berberis-vulg. 200, in water, four doses daily, for one week. Eight days later she returned and reported that she was free from pain that day for the first time, although the pain had been steadily decreasing since she began taking the medicine. The same remedy was continued one week longer. She has remained perfectly well since, and walks as well as she ever did.

Case 3, October 3d, 1897.—Spinster, aged 46. Passing through climacteric.

About two months ago noticed a small swelling in left breast. It increased in size rapidly and about a week ago she visited a prominent surgeon, who examined it and advised immediate removal by the knife. Before submitting to operation desired to consult me to see what Homœopathy could do. The tumor was in the outer and lower segment of the breast about an inch and a half from the nipple. It was about an inch and a half in its longest diameter and one inch in short diameter, somewhat flattened. It was movable, hard, resistant, like a cartilage, and only slightly sensitive to pressure. She had experienced little or no pain in it. Her general health was good. She had a well developed goitre in the left lobe of the thyroid gland, which had existed for many years

Her neck was very thin, though she was plump and well nourished.

Her face flushes very easily, becoming scarlet. She is subject to frequent attacks of tonsilitis, with yellow exudation, which usually begin on the left side. Two attacks, under my care, have been promptly relieved by Lachesis. She is nervous, excitable, and rather irritable. When about twenty years of age she took a great amount of Iodine internally, and it was applied locally to remove the goitre. It failed in its object, but "caused terrible trouble with her stomach."

I prescribed Iodine 45M (Fincke), one dose dry, and S. L. Three days later she came in consternation to show me her breast and report. About forty-eight hours after taking the dose of Iodine the breast began to be painful, with sharp, stinging pains running through it. The breast is swollen, hot, and feels heavy. She fears an abscess is forming. A red spot the size of a silver quarter dollar to the left of the nipple, over the tumor. The nipple is turgid and protruding. The whole glandular structure of the breast appears swollen, and it is very sensitive, but the tumor appears somewhat softer. She feels nauseated and is feverish and apprehensive.

I reassured her, telling her that the disturbance was a part of the curative process set up by the remedy, and would soon pass away, and gave her Sac-lac. to take in water at two-hour intervals. By the next day the pains and swelling began to disappear and the breast resumed its ordinary appearance after about three days, with the tumor growing decidedly softer and smaller. In a little over six weeks no trace of the tumor could be found. No medicine but the one dose of Iodine was given.

Case 4,. July 6th, 1897.—Unmarried man, 32 years of age. Up to present time a healthy man, of herculean build, weighing 210 pounds. Uses neither liquor, tobacco, tea, nor coffee.

On June 19th, 1897, cohabited with a prostitute. Ten days later discovered small chancre in mucus fold of prepuce, back of corona, and near frenum, size of split pea. Examination shows characteristic features of true chancre; induration distinct and clearly defined, surrounding a central erosion or ulcer with yellowish indurated base, which is depressed below surrounding tissue, the sore being only very slightly sensitive to pressure, and painless.

The closest examination failed to elicit but one symptom in addition to the local symptoms. His tongue very plainly showed the imprint of the teeth.

I prescribed Merc-s., CM (Swan), one dose dry, and directed that the parts should be bathed twice daily with pure water, and a small pledget of absorbent cotton applied to the sore.

July 13th.—One week later, reported nothing but a slight itching at site of chancre, which had increased somewhat in size. The yellow base of the ulcer had disappeared and the sore looked clean and red. Sac-lac.

July 20th.—Sore looks rather bluish, and there is now a slight yellowish discharge showing on the cotton. More swelling and slight erosion appears on side of frenum, as if chancre was spreading. Painful on retracting prepuce. Itching slightly. Tongue still indented. Merc-s., CM (Swan), one dose.

July 26th.—Chancre smaller, getting softer, no soreness, no discharge, no concavity—healing. Scalp sensitive to combing. Merc-s., CM (S.), one dose.

August 3d.—Papular eruption, slightly scaly, on scalp, which is still sensitive. Small oval erosion on upper side prepuce, on the skin. Red rash in groins, but no glandular swellings. Tongue still indented. Chancre is clean and growing smaller. Swollen glands on right side, occiput and nape. Merc-v., 45M (Fincke), one dose.

In about ten days the chancre had entirely healed and induration disappeared.

August 23d.—For last ten days has noticed sores in mouth, on edge of tongue and inside lips. Throat feels sore on swallowing and tonsils are swollen and red. Eruption, or rather ulceration, at corners of mouth. Nitric-acid, 45M (Fincke), one dose.

August 28th.—Large sensitive oval ulcer, yellowish base, on right edge of tongue, and smaller one on left side. Tonsils swollen and coated with grayish film of mucus secretion. Throat burns like fire on swallowing. Saliva increased, thick and adhesive. Tongue imprinted. Kali-bi., 45M.

September 1st.—No improvement. Sac-lac.

September 5th.—No improvement; is hoarse, as from a cold. Cough, worse speaking. Burning pain in throat, worse drinking cold water. Ars., 40M (Fincke), one dose.

Improvement followed and continued until October 11th, when increase in throat symptoms demanded a second dose of Ars., 40M, which was given, with benefit, and all went well until January 3d, 1898, when the throat became badly inflamed again. Painful on swallowing, sneezing, and yawning. Tonsils swollen red, with follicular exudation showing in yellowish points, like lid of a pepper-box. Uvula and arch of palate red and swollen. "Cold water burns his throat." Sensation of a lump in the throat, which he can neither swallow nor get up. Desires cold drinks, though they hurt throat. Lac-can., 45M (F.), one dose.

January 17th.—Throat rapidly and steadily improved, and is now nearly well. Sac-lac. About a month later some return of throat symptoms, which were removed by another dose of Lac-can., 45M. I kept him under observation about two months more and discharged him cured.

It so happens that this is the first and only case of syphilis I have ever had in its initial stage and in which absolutely nothing had been done in the way of treatment. The few other cases falling into my hands had always been previously treated by allopathic means, and gave me much trouble. I

was interested in watching the progress of such a case, therefore, under high potencies and single doses. The patient, who had an opportunity to compare his progress with that of a friend who was infected about the same time, was highly delighted at the superiority of homœopathic treatment. I confess, however, that I was disappointed in the result. I had hoped to avoid secondary symptoms, especially as the case seemed such a favorable one for treatment, basing my hope principally on the dictum of Hahnemann, that such would be the result of homœopathic treatment in an uncomplicated case.

### DISCUSSION.

Dr. Pease—I want to ask members of this society if in the treatment of cases of syphilis that have not been tampered with they observed secondary symptoms coming out? I call to mind three cases I have treated, which, I believe, had not been tampered with. There was a very satisfactory and rapid progress of cure of the initial symptom, but there was absolutely no further demonstration of the disease. Dr. Close's case was remarkable any way in that the secondary symptoms appeared and disappeared so rapidly.

Dr. Kimball—Ought there to have been any secondary symptoms after the disappearance of the chancre? In those cases which have come to me first the chancre usually remained some time without healing, and there was considerable discharge for several weeks. I can only recall one case where the secondary symptoms appeared, and they were only slight eruptions on the face and abdomen.

Dr. Close—The man was apparently a magnificent specimen of healthy manhood, yet, about six years previous to this he came to me with the remains of a serious attack of articular rheumatism, and with heart symptoms quite pronounced. I know nothing about the treatment which he underwent except that it was allopathic, but as I studied the case at that time I found a perfect picture of Lachesis, and it very promptly relieved him, removing the remains of rheumatic symptoms, and clearing up the heart symptoms perfectly. I think he only had one dose of Lachesis in the 1000th potency. Probably some other miasm besides syphilis was present in the case of which the rheumatism may have been an expression, but the most patient questioning failed to elicit any other symptoms than the few stated. This would account for the appearance of the secondary symptoms in spite of Mercury, which was certainly indicated, and did good work.

Dr. Pease—Was there any offensive element about the discharges?

Dr. Close—No, there was not.

